# The NutriAct Family Study: a web-based prospective study on the epidemiological, psychological and sociological basis of food choice

**DOI:** 10.1186/s12889-018-5814-x

**Published:** 2018-08-03

**Authors:** Lukas Schwingshackl, Ulrike Ruzanska, Verena Anton, Raphael Wallroth, Kathrin Ohla, Sven Knüppel, Matthias B. Schulze, Tobias Pischon, Johannes Deutschbein, Liane Schenk, Petra Warschburger, Ulrich Harttig, Heiner Boeing, Manuela M. Bergmann

**Affiliations:** 1 NutriAct–Competence Cluster Nutrition Research, Berlin-Potsdam, Germany; 2 Department of Epidemiology, German Institute of Human Nutrition Potsdam-Rehbruecke, Nuthetal, Germany; 3 Department of Psychology, Counseling Psychology, University of Potsdam, Potsdam, Germany; 4 Charité – Universitätsmedizin Berlin, corporate member of Freie Universität Berlin, Humboldt-Universität zu Berlin, and Berlin Institute of Health, Institute of Medical Sociology and Rehabilitation Science, Berlin, Germany; 5 Psychophysiology of Food Perception, German Institute of Human Nutrition Potsdam-Rehbruecke, Nuthetal, Germany; 6 Department of Molecular Epidemiology, German Institute of Human Nutrition Potsdam-Rehbruecke, Nuthetal, Germany; 7 Molecular Epidemiology Research Group, Max Delbrück Center for Molecular Medicine in the Helmholtz Association, Berlin, Germany

**Keywords:** NutriAct family study, Study protocol, Food choice, Determinants

## Abstract

**Background:**

Most studies on food choice have been focussing on the individual level but familial aspects may also play an important role. This paper reports of a novel study that will focus on the familial aspects of the formation of food choice among men and women aged 50–70 years by recruiting spouses and siblings (NutriAct Family Study; NFS).

**Methods:**

Data is collected prospectively via repeatedly applied web-based questionnaires over the next years. The recruitment for the NFS started in October 2016. Participants are recruited based on an index person who is actively participating in the European Prospective Investigation into Cancer and Nutrition (EPIC)-Potsdam study. This index person was asked to invite the spouse, a sibling or an in-law. If a set of family members agreed to participate, access to individualized web-based questionnaires assessing dietary intake, other health related lifestyle habits, eating behaviour, food responsiveness, personality, self-regulation, socio-economic status and socio-cultural values was provided. In the first phase of the NSF, recruitment rates were monitored in detail and participants’ comments were analysed in order to improve the feasibility of procedures and instruments.

**Discussion:**

Until August 4th 2017, 4783 EPIC-Participants were contacted by mail of which 446 persons recruited 2 to 5 family members (including themselves) resulting in 1032 participants, of whom 82% had started answering or already completed the questionnaires. Of the 4337 remaining EPIC-participants who had been contacted, 1040 (24%) did not respond at all, and 3297 (76%) responded but declined, in 51% of the cases because of the request to recruit at least 2 family members in the respective age range. The developed recruitment procedures and web-based methods of data collection are capable to generate the required study population including the data on individual and inter-personal determinants which will be linkable to food choice. The information on familial links among the study participants will show the role of familial traits in midlife for the adoption of food choices supporting healthy aging.

**Electronic supplementary material:**

The online version of this article (10.1186/s12889-018-5814-x) contains supplementary material, which is available to authorized users.

## Background

A high quality diet, composed of abundant amounts of plant-based foods including whole grains, fruits, vegetables, nuts, and legumes is one of the most important factors helping to prevent early death [1, 2] and disability in 21 regions worldwide [3], and is therefore a core part of dietary guidelines [4]. However, a large proportion of men and women worldwide do not adhere to these recommendations [5]. Therefore it is important to understand which determinants are related to a healthy food choice and how these food choices can be promoted.

Food choice in its complexity can only be conceptualized and understood when scientists from different disciplines, i.e. epidemiology, psycho-physiology, psychology, and sociology cooperate to measure, disentangle and understand its determinants [6, 7]. The “Determinants of Diet and Physical Activity” (DEDIPAC) project established recently the “The Determinants of Nutrition and Eating” (DONE) framework of determinants that could influence dietary behaviour [8], which demands transdisciplinary research. Not only individual and interpersonal, but also environmental and policy related factors are related to food choice.

However, it is still not clear how far interpersonal relationships can modify food preferences and food choice and which dynamics of food choice exists during the course of life. There is some evidence that family cohesions, relationships, and networks are involved [9], and that individual dietary behaviour such as restrained eating, food neophobia or cognitive mechanisms are important for food choice [10]. However, the role of the familial environment for food choice with consideration of psycho-physiological, sociological, and psychological determinants has been rarely investigated [11–13].

We hypothesize that food choice is learned in the origin family during childhood, but modified by subsequent partnerships [14].

For this purpose, we selected a study design and data collection methods facilitating the comparison of determinants of food choice across spouses as compared to siblings. The rationale is to observe influences emerging due to sharing the same environment during childhood in contrast to influences emerging by living with a partner who was socialized in a different familial context but share the environment in adulthood. In this paper, we describe the design, the methods, and the data to be collected for the NutriAct Family Study (NFS). We report the first data of participation and of the feasibility of the methods.

## Methods

**Fig. 1:**
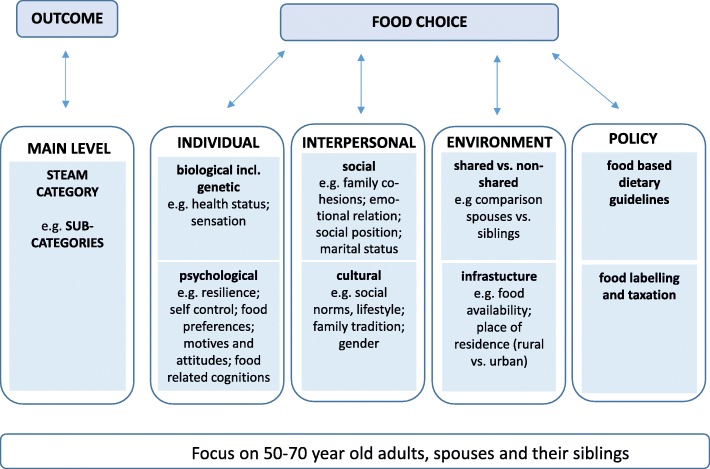
Main dimensions and levels of food choice covered by the NutriAct Family Study (NFS)

The NFS is part of the “NutriAct: Nutritional Intervention for Healthy Aging” cluster, one of the four “Competence Clusters for Nutrition Research” in Germany. Specifically, NutriAct will address nutrition related questions in the context of healthy aging (Fig. 1).

### Study design

In the NFS, we aim to include groups of at least three family members, i. e. to recruit two spouses and at least one sibling of one of the spouses (Fig. 1). The data will be collected prospectively via repeated web-based questionnaires over the next years.

**Fig. 2:**
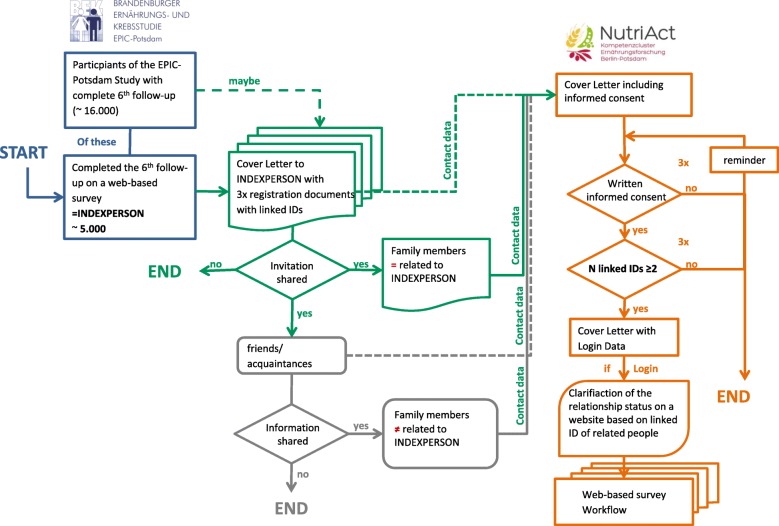
Flow chart of the recruitment strategy of the NutiAct Family Study (NFS) and formal inclusion criteria with participants of the European Prospective Investigation into Cancer and Nutrition (EPIC)-Potsdam study serving as index persons

In total, the study aims to include 3000 men and women in the age range of 50 to 70 years. So far, participants have been recruited based on an index person participating in the European Prospective Investigation into Cancer and Nutrition (EPIC)-Potsdam study [15] who was invited by mail to bring along the spouse and a sibling or an in-law. Eligible were participants who completed the 6th follow-up wave of EPIC-Potsdam (*N* = 16,195), particularly those who filled in the web-based version (*N* = 3990) or agreed to communicate via e-mail (*N* = 793). These 4783 persons were chosen as index-person and received via mail an invitation to participate in the NFS with their family members (Fig. 2).

The mailing to the eligible participants of the EPIC-Potsdam study included a personal letter of invitation with an information brochure about the study, a reply form with a return envelope, and two envelopes containing the same material but a neutral letter of invitation. The two envelopes were to be passed on to family members interested in the study. The reply form included by default the inquiry of contact information and in the case of refusal also for a reason of non-participation. The identifiers of the three reply forms were linked to allow identification of the group members upon return of the forms. All data from the reply forms were entered in the participant’s management software. Next, the potential participants received the informed consent form (IC) by mail. To avoid drop outs due to a prolonged waiting time in case one family member was late with the return of the IC, the group members were mailed the access codes for their questionnaires when at least two of the three ICs were returned (Fig. 2).

The performance of the recruitment procedures were monitored within a pre-phase period of three months (October to December 2016) and the regular mailing lasted from January to March 2017. After that, potential participants were repeatedly reminded by phone and mail.

### Web-based questionnaires – Content

The instruments for the web-based questionnaires were selected according to categories and definitions of the DONE framework. Relevant aspects influencing human eating behaviour and the nutrition from a transdisciplinary perspective have been compiled and operationalized [8]. For our questionnaires, the main levels “individual”, “interpersonal”, and “environment” were determined as relevant; within the levels however, the relevant items were selected by each of the respective experts (i.e. epidemiologists, psychologists, sociologists). For example, health-induced diet or the ability to taste and smell are biological categories on the individual level which are related to food choice; psychological factors are resilience, self-control, food preferences, motives, and attitudes. Interpersonal determinants were sub-divided into the categories “social” and “cultural”, particularly addressing social position, familial values, and traditions. The main level “environment” was also considered in the questionnaire but rather due to questions about the family setting, as well as the character (i.e. urban vs. rural) of the place where the participant was socialized and where he or she lives now.

### The design of the web-based questionnaires

Existing instruments, i.e. questionnaires, inventories, and scales of dietary intake and other lifestyle behaviour, eating behaviour, food responsiveness, personality, self-regulation, socio-economic status, and socio-cultural values were compiled into four coherent parts of one web-based questionnaire each taking one hour at most to be filled in. The participant was given 4 weeks after each questionnaire to start working on the next one. The four web-based questionnaires had been split into sections each of which was saved only after all questions were answered. Only then the next section of the questionnaire was displayed. Exceptions were the third and fourth questionnaire, where psychological and sociological scales contained sensitive questions. Here, the software alerted the participant about unanswered questions but did not require an answer to continue the survey. The implemented instruments were ordered in a way where the workflow went from easy to more complex and from factual to the most private questions. For instance, it is known that applying the scale about personality potentially biases the response to other scales, and therefore these questions were implemented at the very end of the questionnaire.

Information on the validity and reliability of the single instruments are given in the Additional file 1: Table S1. The description of the single instruments and scales included in the web-based questionnaires of the NFS are displayed in Table 1.

**Table 1:** Overview of the instruments and scales implemented in the NutriAct Family Study on Determinants of Food Choice (NFS); Potsdam, Germany

Level	Concept	Instruments and scales	Construct	Applied in questionnaire no.	N items	Short description
INDIVIDUAL	Food responsiveness	Power of Food Scale (PFS)	Appetite	3	15	The PFS assesses the psychological impact of living in food-abundant environments (appetite for palatable foods)
		Short version Behavioural Inhibition Scale (BIS-15)	Approach and avoidance	3	15	The BIS-15 measures on two scales dispositional differences in behavioural approach (BAS-scale) and inhibition (BIS-scale)
		Food Craving Questionnaire (FCQ-T-reduced)	Food craving	3	15	The FCQ assesses craving for a variety of foods covering behavioural, cognitive and physiological aspects of craving
		Reward-based Eating Drive Scale (RED)	Reward-based (over-) eating	4	9	The RED measures the vulnerability to weight-gain associated behaviours such as drive to overeat, lack of control/satiation, preoccupation with food
		Reward-Responsiveness-Scale (RR-Scale)	Reward responsiveness	4	8 + 2 ^a^	The RR-scale assesses the extent to which an individual is sensitive to signals of reward
	Personality	Big 5	Personality	4	16	The scale assesses five personality dimensions: neuroticism, extraversion, openness to experience, compatibility, and conscientiousness
		Resilience	Resilience	4	15	The scale assesses stress, coping ability and, as such, could be an important target of treatment in anxiety, depression, and stress reactions
		Dispositional Optimism	Optimism	4	5	Dispositional optimism has been defined in terms of life engagement and generalized positive outcome expectancies for one’s future
		SEA-K	Social desirability	4	2	The SEA-K measures socially desirable responses
	Eating behaviour	Intuitive Eating Scale-2 (IES-2)	Intuitive eating	4	23	The IES-2 assesses intuitive eating that is eating in line with hunger and satiety cues
		Self-Report Index of Habit Strength (SRHI)	Habit strength	3	12	The SRHI measures habit strength of eating a plant-based diet
		Dutch Eating Behaviour Questionnaire (DEBQ)	External, emotional and restrictive eating	4	30	The DEBQ assesses three different eating styles namely external, emotional and restrictive eating
		Food Neophobia Scale (FNS)	Food Neophobia	4	8	The FNS assesses a reluctance to eat and/or to avoid novel foods
		Dieting	Dieting	2	3	These items asses dieting habits
		Nutrition self-efficacy	Nutrition self-efficacy	4	5 ^a^	These items asses nutrition self-efficacy
	Self-regulation	Short Version of the Self-Control Scale (SCS-K-D)	Self-control	4	13	The SCS-K-D measures general self-control abilities
		Self-Regulation Scale (SRS)	Self-regulation	4	7	The SRS measures general self-regulation skills
		General Self-Efficacy Scale-6 (GSE-6)	Self-efficacy	4	6	The GSE-6 measures general self-efficacy
INDIVIDUAL	Socio-cognitive variables	Willingness to change	Willingness to change	4	1 ^a^	Willingness to change assesses if and when an individual wants to change its nutritional habits in the direction of eating more plant-based foods
		Outcome expectations	Outcome expectations	4	25 ^a^	Outcome expectations ask for the perceived consequences (pros and cons) of eating more plant-based foods
		Risk perception	Risk perception	4	3 ^a^	Risk perception measures the extent to which an individual thinks that not eating plant-based foods can lead to negative health consequences
		Perceived behavioural control	Perceived behavioural control	4	5 ^a^	Perceived behavioural control measures the extent to which eating more plant-based foods is within one’s control
		Norms	Norms	4	4 ^a^	These items asses the perceived pressure of family and friends to eat more plant-based foods
		Attitudes	Attitudes	4	10 ^a^	These items asses the individual’s attitudes towards eating more plant-based foods
	Lifestyle	Physical activity	Physical activity	2	75	Development of an improved physical activity index, which is able to categorize study participants into activity categories but may also be used as a continuous measure that reflects physical activity and sedentary time
	Life situation	Dietary change due to illness	Dietary change	1	19	These items asses dietary changes due to illness
		Lifetime Alcohol and Smoking	Lifetime	2	5 + 1	These items asses alcohol intake and smoking
		Quality of life (SF-8)	Quality of life	2	8	Health-related quality of life is an individual’s or a group’s perceived physical and mental health over time
		Amsterdam Instrumental Activities of Daily Living Questionnaire (A-IADL)	Instrumental Activities of Daily Living	2	6	The A-IADL-Q is a disease-specific IADL questionnaire, aimed at measuring IADL problems in early dementia
		Socio-economic and sociodemographic status	Individual and micro environment socio-economic status	1	19	These items assess the personal socio-economic status as well as socio-economic variables of the micro environment (i.e. background family, partner)
INTERPERSONAL	Social values orientation	social influence and nutrition	social influence and nutrition	3	14	Development of a short item list to evaluate familiar taste and cooking preferences in relation to the actual social setting
		nutrition and lifestyle habits	eating values	3	10 ^a^	The instrument is based on BZgA survey and evaluates different nutritional orientations regarding daily food habits
	Socio-cultural habits	Human Value Scale (HVS)	Human Value Scale (HVS)	3	21	The Human Values Scale (HVS) of the European Social Survey (ESS) is a measure that classifies respondents according to ten basic value orientations: achievement, benevolence, conformity, hedonism, power, security, self-direction, stimulation, tradition, and universalism
		Construct of cultural activities	Cultural activities	3	40	The questionnaire evaluates how cultural assets influence the individual lifestyle relative to other socio-demographic factors
	Familial shaping	Familial eating habits	Familial eating habits	1	15	These items asses familial eating habits and eating traditions
		Familial attitudes	Familial attitudes	1	10 ^a^	These items asses the familial attitudes towards eating more plant-based foods
ENVIRONMENTAL	Familial network	Intimate Relationships and Family Dynamics	Family relations: Contact, emotional closeness, travel-time distance	2	10 ^a^	Short scale based on pairfam survey, evaluates familial relationships and emotional closeness
		Number of siblings	Number of siblings	1	1	This item assesses the number of siblings
	Place of residence	Rural and urban living environment	Place of residence	3	4	These items asses the place of residence in respect to rural and urban areas
OUTCOME	Dietary intake	Food Frequency Questionnaire	Habitual diet	1	188	For the repeated dietary assessment in the European Prospective Investigation into Cancer and Nutrition (EPIC)-Potsdam Study, a simple FFQ with low respondent burden was developed to measure dietary intake
		24 h food list	Habitual diet	Single questionnaires additional to the main survey	90	To assess dietary intake a short 24-h food list based on German survey data was developed. In a second step, evaluating the feasibility and acceptability of repeated applications of this tool by study participants of the pretest of the German National Cohort study during a 6-month period

^a^ modified version

As primary outcome, usual **dietary intake** was assessed using a food frequency questionnaire (FFQ), and four times a 24-h food lists (24hFL) applied on a random day over a period of 12 months after the first time of logon by the participant. The 24hFL is a simplified web-based questionnaire asking whether a specific food was consumed on the previous day without specifying meal time or portion sizes [16]. **Alcohol consumption** was assessed by a series of comprehensive questions taking into account lifetime alcohol use, binge drinking, frequency of alcohol consumption, and alcohol drinking with meals [17]. Assessment of **physical activity** was based on an Improved Physical Activity Index which was evaluated and validated in the EPIC-Potsdam study [18]. Information on health-related **quality of life** was collected using the SF-8 questionnaire [19], and the Amsterdam Instrumental activities of daily living (IADL) inventory which measures the degree of self-determined life [20]. Various scales on **eating behaviour** [21, 22] including intuitive eating [23, 24], and self-efficacy and self-regulation as essential psychological concepts related to eating behaviour [25–27]. Accordingly, food responsiveness – essentially the appeal of or desire towards food – was an extension in these psychological constructs which is why scales of power of food [28], food craving [29], and reward-based eating [30] were also incorporated in the web-based questionnaires. The classification of the personality was based on the Big 5 [31], resilience [32], and dispositional optimism [33]. Socio-cognitive variables were included as well as stages of change and familial eating habits and eating values [34, 35].

Social status was assessed by standard questions on socioeconomic characteristics [36–38]. The sociocultural background was furthermore operationalized by lifestyle orientations, cultural values, and cultural activities (such as visits of cultural events, friends, museums, or playing music) [36, 39–42]. Additionally, specific orientations regarding partnerships and the origin family on eating and taste were taken into account by self-constructed questionnaires (e.g. familiar taste, family traditions, ambience, cooking preferences, and general diet and consumer orientations). To evaluate the social relationships, the environment and family setting and their influence on food choice, questionnaires measuring frequency of contact to family members, and emotional closeness were used [43–46]. The NFS as a prospective longitudinal study also enables analyses stratified by different social transition phases. These include the transition into retirement, changes in occupational status (e.g., employed to unemployed or full time employment to part time) as well as changes in the family context (widowhood or separation), children leaving their homes, or a change of residence. Finally, two items were included to measure the tendency of responding according to a “social desirability” [47].

The NFS was approved by the ethical committee of the Medical Association of the State of Brandenburg in Cottbus (Germany) (EK der LÄKB S 21(a)/2015).

### Statistical analysis

Descriptive statistics of mailed invitations, returned response forms with reasons of non-participation, proportions of participation and response to questionnaires including comments given in the open fields for the time period from October 1st 2016 to August 25th 2017 were generated. In order to investigate the mood of the participants and to identify potential problems with the questionnaire, the open comments given at the end of each of the four parts of the questionnaire were analysed with a content analysis which classifies comments according to four major areas (methods, conditions of living or health, dietary habits, other comments). The areas with their respective sub-categories (Table 2) have been defined in an iterative process of reviewing of one author (MMB) followed by a review and a second independent coding (SG).

**Table 2:** Number of comments given by participants of the NutriAct-Family study (NFS) in the open field at the end of each of the four web-based questionnaires

	N	Questionnaire 1	Questionnaire 2	Questionnaire 3	Questionnaire 4
Number of participants who wrote a comment	246	166	54	23	3
Number of categorized comments	430	146	134	83	67
Categories:	N	%	%	%	%
Questionnaires, questions, response models or method					
Additional information	63	36	48	14	2
Technical or operation issues	35	34	9	17	40
Complains (e.g. about burden)	41	22	7	27	44
Issues of recruitment procedures	5	40	20	0	40
Conditions of living or health					
Health status	74	18	72	5	5
Implications due to conditions of family members	8	25	75	0	0
Mental aspects	21	0	48	43	9
Crucial working conditions	14	43	36	21	0
Dietary habits					
Specific diet	22	36	32	18	14
Use of specific products	40	96	0	2	2
Values regarding nutrition	31	26	3	68	3
Avoidance of certain food	10	80	0	0	20
Other comments					
Wish of return of results	6	50	0	0	50
“No comment”	6	33	33	17	17
Positive reflection on participation	6	50	17	0	33
Philosophy or history of life	26	15	43	23	19
Humorous reflections	22	23	5	36	36

Finally, the feasibility of procedures and instruments as well as the success of recruitment and cooperation of the participants recruited were evaluated based on this descriptive analysis.

### Status of the study

#### Number of study participants

Until 4th of August 2017, 1032 study participants were recruited by 446 index persons (of the 4783 invited) who participate in the EPIC-Potsdam study (Table 3). The numbers of family members recruited by each index person varied from 2 to 5 (including themselves), with a high proportion of two spouses with no sibling yet. Overall, 9% of the eligible EPIC-Potsdam participants were able to recruit mainly the spouse but to lesser extent also a sibling.

**Table 3:** Number of Participants according to group size who signed the informed consent, are online or completed the first online-survey of the NutriAct Family Study on Determinants of Food Choice (NFS) until August 4, 2017; Potsdam, Germany

Group size	Total N Families	Total N Persons	Not yet online N Persons	Started online survey^a^ N Persons	Completed all 4 questionnaires N Persons
Two siblings (no spouse)	10	20	6	10	4
Two spouses (no sibling)	311	622	144	250	228
and 1 sibling	112	336	30	138	168
and 2 siblings	11	44	4	12	28
and 3 siblings	2	10	5	5	0
Total	446	1032	189	415	428

^a^ Access to the online-questionnaires is mailed when 2 persons in a group have returned the signed informed consent by mail. The completion of the group is then subject to reminding activities by phone and e-mail. If finally the third person fails to join, the group will nevertheless be accepted

**Fig. 3:**
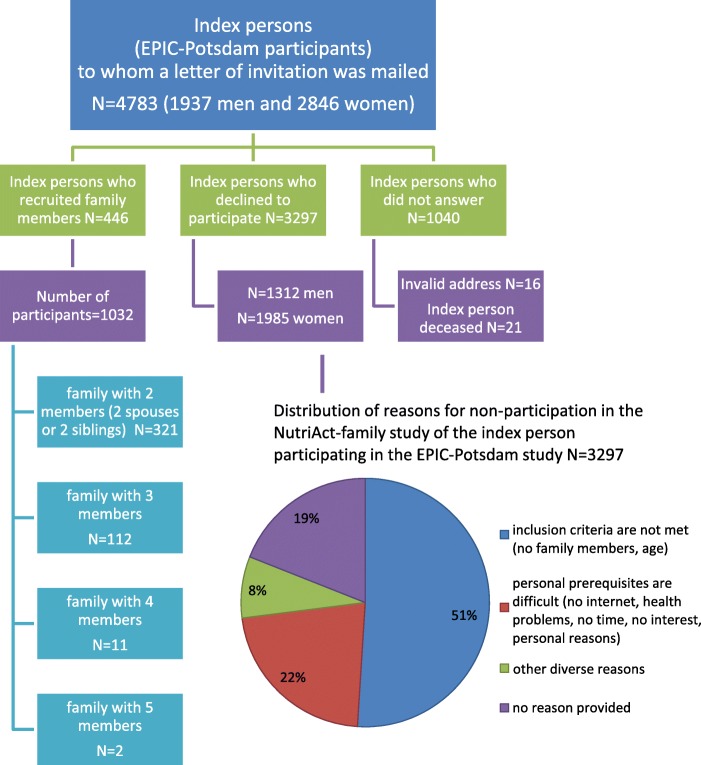
Reasons for non-participation of index persons who are participants of the European Prospective Investigation into Cancer and Nutrition (EPIC)-Potsdam study (Status as of August 4th, 2017) and distribution of reasons for non-participation in the NutriAct-family study of the index person participating in the EPIC-Potsdam study (Status as of August 4th, 2017)

Of the invited EPIC-Potsdam study participants (*N* = 4783), 91% (*N* = 4337) refused to participate in the NFS of which 76% (*N* = 3297) replied the form or answered via phone and 24% (*N* = 1040) did not respond at all (Fig. 3). In the replied forms, the most prominent reported reason for non-participation was the difficulty to recruit a spouse and a sibling in the required age range (51%). A fifth of the replies disclosed no reason for decline and another fifth of the statements referred to personal prerequisites such as no time, health problems or no internet.

#### Response behavior

Of the 1032 participants, 41% had finished all four questionnaires, 40% started working on their questionnaires, and about 18% of the participants had received their login-data but did not go online yet (Table 3). Of those who completed all four questionnaires (*N* = 428), 57% wrote at least one comment (Table 2). The majority of comments were given at the end of the first and the second questionnaire (34 and 31%) and regarded mainly information on individual diet, familial nutritional habits, family relations, familial socio-economic status, physical activity or alcohol consumption. Comments in general concerned the methods (e.g. the way questions and answers were designed (34%)), individual and familial living or health conditions (27%), dietary habits (24%), or other matters (15%). Most of the comments given in the first two questionnaires were additional information to single questions and explanations of answers or on the individual health status. But also 10% (41 of 430) of the comments were complaints about the burden to answer that many questions with the highest proportion (44%) in questionnaire 4, compared to 22%, 7%, and 27% in questionnaires 1, 2, and 3 respectively. Questionnaire 4 comprised most of the psychological scales.

## Discussion

The present paper described the design and methods of the NFS and reported data of the first period of recruitment of participants and their general acceptance of the procedures of recruitment and data collection. In the first period, EPIC-Potsdam study participants were chosen as index persons with the aim to recruit family members: the spouse and a sibling of one of the spouses (family triple). Nine percent of the index persons succeeded to recruit family members for the family cohort. The most prominent reason for non-participation included not fulfilling the inclusion criteria of a family triple. The lack of personal prerequisites (i.e. no time, no interest, no internet, health problems, and personal reasons) was only relevant for about a fifth of the participants who reported a reason. Though the yield of the mailing of invitations to EPIC-Potsdam participants was low, in principle the recruitment strategy and the application of the web-based questionnaires were proven to be feasible by the number of successfully recruited participants.

Internationally, there are several studies under way where the study population consists of family members or descendants, i.e. linked persons. Among the most important are: a) The Long Life Family Study consisting of index persons ages 90 years and older and their families selected for research on exceptional familial longevity in the United States and Denmark [12], b) the Heinz Nixdorf Recall “MehrGenerationenStudie” in Essen recruiting offspring of participants of the Heinz Nixdorf Recall study (comparable to the Framingham Heart study offspring cohort) with the focus on research on risk factors for cardiovascular diseases [48], and c) the Swedish LifeGene study aiming at developing a prospective, population-based cohort as a resource for research in biomedicine as well as behavioural and social sciences, including also descendants of participants as family members [13]. These three studies are based on multigenerational populations with the focus on biomedical outcomes. There is no comparable study to ours aiming at disentanglement of the roles of personal and interpersonal aspects of food choice for healthy aging based on a study population that includes familial links across the same generation. The recruitment activities will therefore be continued until the goal of a study population consisting of 3000 participants is reached. Since the pool of all eligible participants of the EPIC-Potsdam study as an index person has been exploited, we will continue recruitment from other sources of population, e.g. by advertisement in newspapers, on internet platforms, in clubs, or by popular science talks in organisations where people in the required age range are represented.

The transdisciplinary study will offer the opportunity to analyse the convergence or divergence of behaviours and attitudes between spouses as compared to siblings. The collection of data from multiple research perspectives allows examining their relative influence on food choice and finally dietary intake as a central outcome. To asses this outcome, novel methods of estimation of dietary intake will be applied [49]. Psychological scales and sociological inventories implemented in the questionnaires will be analysed by established bivariate and multivariate statistical methods, novel approaches, and new statistical methods to inter-correlate scales and scores with nutritional data, e.g. by applying multi-level analysis, will yield new insights into possible mechanisms of intrapersonal, interpersonal and environmental determinants of food choice.

For instance, one interesting psychological construct is “intuitive eating”, an adaptive eating style [23]. First results in French adults have revealed its association with healthier food choices [50]. Another construct is eating behaviour in a wider sense as part of the overall lifestyle pattern and as such of the habits of daily life [51–55]. Our data will be able to provide insight in how eating behaviour and lifestyles among family members are linked to the possibility to change –e.g. as a function of the micro- and macro-social environment, familial relationships, values and lifestyle orientations [56]. The micro-social environment regards specifically the interpersonal level within the family setting and the macro-social environment including aspects such as social position, cultural factors, and place of living. Furthermore, to identify sensitive spots which may be able to trigger changes in food choice, it seems necessary to investigate the potential for change of the family setting and personal relationships.

Through the identification of the mechanisms of daily food choice in the middle aged population, we aim to form the basis of a long term knowledge-based strategy to newly establish or maintain a healthy diet during the process of aging. One anticipated outcome of the NutriAct project will be the development and enforcement of food-based dietary guidelines to define a healthy diet as part of a healthy lifestyle embedded in the familial setting in the middle ages.

The strengths of the NFS include the transdisciplinary working group which contributed disciplinary expertise and tools to achieve a coherent web-based questionnaire. Another strength is the standardized workflow for all participants, which will facilitate the comparison of determinants of food choice across familial links. The web-based questionnaire allows gradually collecting, saving, and processing large amounts of data at reduced logistic burden and cost. A well-known study, taking advantage of these features, is the NutriNet-Santé Study, which started in France in 2009 with the aim to recruit 300,000 adult participants 18 years and older [57], and included approximately 100,000 participants [58].

Choosing the active members of the EPIC-Potsdam cohort as index persons for a new study has a clear advantage. EPIC-Potsdam participants have been committed during the follow-up for decades and are experienced with paper and web-based questionnaires.

However, the exploitation of this study population has the disadvantage that a positive selection cannot be counteracted. Additionally, the enrolment in the NFS generally depends on the cooperation of family members which is per se depending on intact familial relationships, the survival of family members, the existence of siblings and the ability to use a computer and the internet. These points are clear limitations because it is well established that unhealthy behaviours accumulate in families with dysfunctional relationships [59, 60].

The developed recruitment procedures and web-based methods of data collection are feasible to generate the base for research on individual, interpersonal and environmental determinants of food choice. The information on familial links offers the unique potential to disclose the role of familial relationships in the middle-age group for the adoption of food choices which support healthy aging.

## Additional file

Additional file 1: **Table S1.** Detailed description including reliability and validity of the instruments and scales implemented in the NutriAct Family Study on Determinants of Food Choice (NFS); Potsdam, Germany. (PDF 307 kb)

## Abbreviations

24hFL: 24-h food lists
DEDIPAC: Determinants of Diet and Physical Activity
DONE: The Determinants of Nutrition and Eating
EPIC: European Prospective Investigation into Cancer and Nutrition
FFQ: Food frequency questionnaire
IADL: Amsterdam Instrumental activities of daily living
IC: Informed consent
NFS: NutriAct Family Study
