# Supplementary material for: The NutriAct Family Study: a web-based prospective study on the epidemiological, psychological and sociological basis of food choice
Source: BMC Public Health. 2018 Aug 3;18:963. doi: 10.1186/s12889-018-5814-x (PMC6090749; doi:10.1186/s12889-018-5814-x)
Supplement: Supplementary file 1 — Table S1. Detailed description including reliability and validity of the instruments and scales implemented in the NutriAct Family Study on Determinants of Food Choice (NFS); Potsdam, Germany. (PDF 307 kb) [file 12889_2018_5814_MOESM1_ESM.pdf]

Main level	Cluster	Questionnaires	Reliability and validity	References
**INDIVIDUAL**				
	**Food responsive-ness**	Power of Food Scale (PFS)	- Item-total correlations from .50 to .73<br>- Cronbach's alpha = .91<br>- Test-retest reliability (4 months): r = .77<br>- Validity: significantly related to self-report measures of restraint, disinhibition, hunger, emotional eating and external eating (r = .30 - .66)	Lowe MR, Butryn ML, Didie ER, Annunziato RA, Thomas JG, Crerand CE, Ochner CN, Coletta MC, Bellace D, Wallaert M, Halford J. The Power of Food Scale. A new measure of the psychological influence of the food environment. Appetite 2009, 53(1): 114-118. doi: 10.1016/j.appet.2009.05.016.
		Short version Behavioural Inhibition Scale (BIS-15)	- Cronbach's alpha = .78 (BIS) / .81 (BAS)<br>- Split-half reliability = .79 (BIS) / .82 (BAS)	Strobel A, Beauducel A, Debener S, Brocke B. Eine deutschsprachige Version des BIS/BAS-Fragebogens von Carver und White. Zeitschrift für Differentielle und Diagnostische Psychologie 2001, 22: 216-227.
		Food Craving Questionnaire (FCQ-T-reduced)	- Cronbach's alpha = .94<br>- 3-week retest-reliability: r > .80<br>- Correlations in 2 studies with BMI (r = .18 and r = .26) and self-perceived dieting success (r = -.40 and r = -.35)	Meule A, Hermann T, Kubler A. A short version of the Food Cravings Questionnaire-Trait: the FCQ-T-reduced. Front Psychol 2014, 5: 190.
		Reward-based Eating Drive Scale (RED)	- Cronbach's alpha = .81 - .92<br>- Correlation with BMI (r = .34-.35)	Epel ES, Tomiyama AJ, Mason AE, Laraia BA, Hartman W, Ready K, Acree M, Adam TC, St Jeor S, Kessler D. The reward-based eating drive scale: a self-report index of reward-based eating. PLoS One 2014, 9(6):e101350.
		Reward-Responsiveness-Scale (RR-Scale)	- In a student sample: Cronbach's alpha = .71 - .81<br>- EFA / CFA: one factor solution<br>- Validity: negative relationship to sensitivity to punishment; positive relationship to life satisfaction and reward	van den Berg I, Franken IHA, Muris P. A new scale for measuring reward responsiveness. Front Psychol. 2010;1:239. doi:10.3389/fpsyg.2010.00239.

	**Personality**	Big 5	- Cronbach's alpha = .53 - .74	Gerlitz JV, Schupp J. Zur Erhebung der Big-Five-basierten Persönlichkeitsmerkmale im SOEP. Dokumentation der Instrumententwicklung BFI-S auf Basis des SOEP-Pretests 2005
		Resilience	CD-RISC:<br>- Cronbach's alpha = .89<br>- Test-retest reliability demonstrated a high level of agreement, with an intraclass coefficient of = .87<br>RS-25:<br>- Cronbach's alpha = .94<br>RS-13:<br>- Test-retest reliability = .61<br>BRCS:<br>- Cronbach's alpha = .76 (follow-up)<br>- Test-retest correlation = .71	Connor KM, Davidson JR. Development of a new resilience scale: the Connor-Davidson Resilience Scale (CD-RISC). Depress Anxiety. 2003;18:76-82. Epub 2003/09/10. doi: 10.1002/da.10113.<br><br>Leppert K,Koch B, Brähler E, Strauß B. Die Resilienzskala (RS) – Überprüfung der Langform RS-25 und einer Kurzform RS-13. Klinische Diagnostik und Evaluation 2008; 1: 226-243.<br><br>Sinclair VG, Wallston KA. The Development and Psychometric Evaluation of the Brief Resilient Coping Scale. Assessment. 2004; 11: 94-101. doi: 10.1177/1073191103258144.
		Dispositional Opti mism	- Moderate internal consistency (Cronbach's alpha = .62 (1985) / .65 (1990) / .71 (1995) and .63 (2000)	Giltay EJ, Kamphuis MH, Kalmijn S, Zitman FG, Kromhout D. Dispositional optimism and the risk of cardiovascular death: the Zutphen Elderly Study. Arch Intern Med. 2006; 166: 431-6. doi: 10.1001/archinte.166.4.431.<br><br>Hinz A, Schumacher J, Albani C, Schmid G, Brähler E. Bevölkerungsrepräsentative Normierung der Skala zur Allgemeinen Selbstwirksamkeitserwartung. Diagnostica. 2006; 5: 26-32. doi: doi:10.1026/0012-1924.52.1.26.
		SEA-K	- Cronbach's alpha = .63	Satow L. Skala zur Erfassung von Testverfälschung durch positive Selbstdarstellung und sozialerwünschte Antworttendenzen (SEA). Psychomeda Discussion Paper.
	**Eating behaviour**	Intuitive Eating Scale-2 (IES-2)	- Cronbach's alpha = .85 - .89<br>- Test-retest reliability after 3 weeks: r = .88 for women, r =.92 for men	Ruzanska UA, Warschburger P. Psychometric evaluation of the German version of the Intuitive Eating Scale-2 in a community sample. Appetite. 2017;117:126–34. doi:10.1016/j.appet.2017.06.018.

			- evidence for convergent and divergent validity by its correlations with psychological, health and eating-related variables	Tylka TL, Kroon Van Diest AM. The Intuitive Eating Scale-2: Item refinement and psychometric evaluation with college women and men. J Couns Psychol. 2013;60:137–53. doi:10.1037/a0030893.
		Self-Report Index of Habit Strength (SRHI)	- Cronbach's alpha (habit strength of a general healthy eating behaviour) = .92 - .95<br>- Validated for several nutrition and physical activity behaviours	Verplanken B, Orbell S. Reflections on past behavior: A self-report index of habit strength. Journal of Applied Social Psychology. 2003;33:1313–30.
		Dutch Eating Behaviour Questionnaire (DEBQ)	- Cronbach's alpha in a representative sample of the German population: .89 (external eating); .92 (restrained eating); .94 (emotional eating)<br>- Replication of the 3-factor structure in EFA and CFA<br>- Evidence for validity: group differences regarding gender, BMI and age	Grunert SC. Ein Inventar zur Erfassung von Selbstaussagen zum Ernährungsverhalten. Diagnostica. 1989;35:167–79.<br><br>Nagl M, Hilbert A, Zwaan M de, Braehler E, Kersting A. The German Version of the Dutch Eating Behavior Questionnaire: Psychometric Properties, Measurement Invariance, and Population-Based Norms. PLoS ONE. 2016;11:e0162510. doi:10.1371/journal.pone.0162510 .<br><br>van Strien T, Frijters JER, Bergers GPA, Defares PB. The Dutch Eating Behavior Questionnaire (DEBQ) for assessment of restrained, emotional, and external eating behavior. Int. J. Eat. Disord. 1986;5:295–315. doi:10.1002/1098-108X(198602)5:2<295::AID-EAT2260050209>3.0.CO;2-T .
		Food Neophobia Scale (FNS)	- Cronbach's alpha in a student sample = .88<br>- Test-retest reliability: r = .82 - .91<br>- Behavioural validation studies demonstrated that test scores predicted behaviour in laboratory food selection situations<br>- Correlations with trait anxiety,	Pliner P, Hobden K. Development of a scale to measure the trait of food neophobia in humans. Appetite. 1992;19:105-20. doi:10.1016/0195-6663(92)90014-W.<br><br>Ritchey PN, Frank RA, Hursti U-K, Tuorila H. Validation and cross-national comparison of the food neophobia scale (FNS) using confirmatory factor analysis. Appetite. 2003;40:163-73. doi:10.1016/S0195-6663(02)00134-4.

			age, experience seeking subscale (of the sensation seeking scale), general neophobia, general familiarity and experience with unusual foods<br>- Validation in different countries	
		Dieting	N.A.	Ruzanska UA, Warschburger P. Psychometric evaluation of the German version of the Intuitive Eating Scale-2 in a community sample. Appetite. 2017;117:126–34. doi:10.1016/j.appet.2017.06.018.
		Nutrition self-efficacy	- Cronbach's alpha = .87<br>- Evidence for validity	Schwarzer R, Renner B. Health-specific self-efficacy scales. 2009. https://userpage.fu-berlin.de/health/healself.pdf
	**Self-regulation**	Short Version of the Self-Control Scale (SCS-K-D)	- Cronbach's alpha in different samples = .78 - .80<br>- Test-retest-reliability (7 weeks) = .82<br>- Validity demonstrated by its significant associations with self-regulation, life satisfaction, self-esteem and procrastination<br>- One factor structure (EFA / CFA)	Bertrams A, Dickhäuser O. Messung dispositioneller Selbstkontroll-Kapazität. Diagnostica. 2009;55:2-10. doi:10.1026/0012-1924.55.1.2.
		Self-Regulation Scale (SRS)	- Cronbach's alpha = .76<br>- Test-retest-reliability = .73<br>- One factor structure (CFA)<br>- Validated in different languages and significant associations with e.g. general perceived self-efficacy and proactive coping	Luszczynska A, Diehl M, Gutiérrez-Doña B, Kuusinen P, Schwarzer R. Measuring one component of dispositional self-regulation: Attention control in goal pursuit. Personality and Individual Differences. 2004;37:555-66. doi:10.1016/j.paid.2003.09.026.
		General Self-Efficacy Scale-6 (GSE-6)	- Cronbach's alpha = .79 (non-clinical sample) / .86 - .88 (clinical sample)<br>- Validity by its significant associations with e.g. depression,	Romppel M, Herrman-Lingen C, Wachter R, Edelmann F, Düngen H-D, Pieske B, Grande G. A short form of the General Self-Efficacy Scale (GSE-6): Development, psychometric properties and validity in an intercultural non-clinical sample and a sample of patiens at risk for heart failure. GMS Psycho-Social-Medicine. 2013;10:1–7.

			anxiety, mental health and social support	
	**Socio-cognitive variables**	Willingness to change	- Validity of staging measures has been supported in multiple studies<br>- Convergent validity: self-reported motivational stage status for eating a diet low in fat was consistent with motivation, decision-making and coping activities of women	DiClemente CC, Delahanty JC, Havas SW, van Orden OR. Understanding self-reported staging of dietary behavior in low-income women. J Health Psychol. 2015;20:741–53. doi:10.1177/1359105315580213.
		Outcome expectations	- Cronbach's alpha= .72 (Pros), .56 (Cons)<br>- Validity: nutritional intention is predicted by different socio-cognitive factors, such as outcome expectancies	Lippke S, Ziegelmann JP, Schwarzer R, Velicer WF. Validity of stage assessment in the adoption and maintenance of physical activity and fruit and vegetable consumption. Health Psychol. 2009;28:183–93. doi:10.1037/a0012983.<br><br>Renner B, Schwarzer R. The motivation to eat a healthy diet: How intenders and nonintenders differ in terms of risk perception, outcome expectancies, self-efficacy, and nutrition behavior. Polish Psychological Bulletin. 2005;36:7–15.<br><br>Renner B, Hahn, A, Schwarzer R. Berlin Risk Appraisal and Health Motivation Study. 1996. http://userpage.fu-berlin.de/gesund/brahms/inhaltsverzeichnis.htm
		Risk perception	- Cronbach's alpha = .87 (community sample)<br>- Validity: Risk perception is important in intention formation for individuals without intention<br>- Nutritional intention is predicted by different socio-cognitive factors, such as risk perception	Renner B, Schwarzer R. The motivation to eat a healthy diet: How intenders and nonintenders differ in terms of risk perception, outcome expectancies, self-efficacy, and nutrition behavior. Polish Psychological Bulletin. 2005;36:7–15.

		Perceived behavioural control	- Cronbach's alpha = .73 and .74 (health promotion clinic attendees)<br>- Test–retest reliability: r = .53<br>- Convergent validity: PBC is associated with healthy eating behaviour, fruit and vegetable intake, behavioural intention, attitudes, perceived past behaviour and subjective norm	Conner M, Norman P, Bell R. The theory of planned behavior and healthy eating. Health Psychology. 2002;21:194–201. doi:10.1037//0278-6133.21.2.194.
		Norms	- Test–retest reliability: r = .38<br>- Convergent validity: Subjective norm is associated with attitudes, behavioural intention and PBC	Conner M, Norman P, Bell R. The theory of planned behavior and healthy eating. Health Psychology. 2002;21:194–201. doi:10.1037//0278-6133.21.2.194.<br>Manstead ASR, Eekelen SAM. Distinguishing Between Perceived Behavioral Control and Self-Efficacy in the Domain of Academic Achievement Intentions and Behaviors. J Appl Social Pyschol. 1998;28:1375–92. doi:10.1111/j.1559-1816.1998.tb01682.x.
		Attitudes	- Cronbach's alpha = .77 - .86<br>- Test–retest reliability: r = .51<br>- Convergent validity: Attitudes are associated with healthy eating behaviour, fruit and vegetable intake, behavioural intention, PBC, perceived past behaviour and subjective norms	Conner M, Norman P, Bell R. The theory of planned behavior and healthy eating. Health Psychology. 2002;21:194–201. doi:10.1037//0278-6133.21.2.194.<br><br>Bruijn G-J de, Kremers SPJ, Vet E de, Nooijer J de, van Mechelen W, Brug J. Does habit strength moderate the intention–behaviour relationship in the Theory of Planned Behaviour?: The case of fruit consumption. Psychology & Health. 2007;22:899–916. doi:10.1080/14768320601176113.
	**Lifestyle and life situation**	Physical activity	- The correlations with accelerometer ranged r = .40 – .43; with physical activity energy expenditure r = .33 – .40<br>- In non-working participants, r = .34 for accelerometer; r = .29 for physical activity energy expenditure	Wientzek A, Vigl M, Steindorf K, Brühmann B, Bergmann MM, Harttig U, Katzke V, Kaaks R and Boeing H: The improved physical activity index for measuring physical activity in EPIC Germany. PLoS One 9, 2014: e92005

		Dietary change due to illness	N.A.	self-established
		Lifetime Alcohol and Smoking	N.A.	Dawson DA. Methodological issues in measuring alcohol use. Alcohol Research Health, 2003; 27: 18-29.<br><br>Greenfield TK, & Kerr WC. Alcohol measurement methodology in epidemiology: recent advances and opportunities. Addiction, 2008; 103: 1082-99.<br><br>Rehm J. Measuring quantity, frequency, and volume of drinking. Alcohol Cliniclal Expert Research, 1998; 22: 4S-14S.
		Quality of life (SF-8)	- Test-retest reliability: r = .61 for physical component scoring; r = .68 for mental component scoring (Roberts et al. 2008)	Ware, J. E., & GlaxoSmithKline. How to score and interpret single-item health status measures: A manual for users of the of the SF-8 health survey : (with a supplement on the SF-6 health survey). 2001, Lincoln, RI: QualityMetric, Inc.
		Amsterdam Instrumental Activities of Daily Living Questionnaire (A-IADL)	- Diagnostic accuracy based on the AUC was 0.75 (95% confidence interval [CI]: 0.70-0.81)	Sikkes SA, Pijnenburg YA, Knol DL, de Lange-de Klerk ES, Scheltens P, Uitdehaag BM. Assessment of instrumental activities of daily living in dementia: diagnostic value of the Amsterdam Instrumental Activities of Daily Living Questionnaire. J Geriatr Psychiatry Neurol. 2013; 26: 244-50. doi: 10.1177/0891988713509139.
		Socio-economic and sociodemographic standard	N.A.	Ehling, M., Hoffmeyer-Zlotnik, J. H. P., Quitt, H., von der Heyde, C., & Bosch, V. 2004. Demografische Standards. Statistisches Bundesamt. Available for download at http://www. gesis. org/Methodenberatung/Untersuchungsplanung/Standarddemografie .Hoffmeyer-Zlotnik, J. H., Hess, D., & Geis, A. J. (2004). Computerunterstützte Vercodung der International Standard Classification of Occupations (ISCO-88). ZUMA Nachrichten, 28(55), 29-52.
		Rural and urban living environment	N.A.	Baumann, H., Schulz, S., & für Sozialwissenschaften, G. D., 2015. ALLBUS 2014–Variable Report. GESIS Daten.// Petermann, Sören. Soziale Vernetzung städtischer und ländlicher Bevölkerungen am

				Beispiel der Stadt Halle: Abschlussbericht und Codebuch. Martin-Luther-Universität Halle-Wittenberg, Institut für Soziologie, 2001.
**INTER-PERSONAL**				
	**Social values orientation**	Social influence and nutrition	N.A.	Schenk L, Deutschbein J, Anton V, 2017, Institut für Medizinische Soziologie und Rehabilitationswissenschaft Charité Universitätsmedizin Berlin (Self contructed Items)
		Nutrition and lifestyle habits	N.A.	Gerhards J & Rössel J, 2003 (BZGA) Forschung und Praxis der Gesundheitsförderung, Band 20: Das Ernährungsverhalten Jugendlicher im Kontext ihrer Lebensstile. Eine empirische Studie. Köln: BZgA, 2003. ISBN 3-933191-79-3
	**Socio-cultural habits**	Human Value Scale (HVS)	- Cronbach's alpha for the Human Values Scale total: Security = 0.63; Conformity = 0.56; Tradition = 0.40; Benevolence = 0.65; Universalism = 0.60; Self Direction = 0.50; Stimulation = 0.64; Hedonism = 0.67; Achievement = 0.72; Power = 0.48	Schwartz, SH., Breyer, B., & Danner, D, 2015, Human Values Scale (ESS). Zusammenstellung sozialwissenschaftlicher Items und Skalen. doi:10.6102/zis234 (Online Fragebogen 3 S.10-13)
		Construct of cultural activities	N.A.	Blom, A G, Bossert, D; Gebhard, F; Herzing, J; Krieger, U, 2014; SFB 884 ´Political Economy of Reforms´ (2016): German Internet Panel, Wave 14 (November 2014). GESIS Data Archive, Cologne. ZA5925 Data file Version 2.0.0, doi:10.4232/1.12620
	**Familial network**	Intimate Relationships and Family Dynamics	- Cronbach's alpha = 0.82<br>- Nearly all measurement models fit very well (RMSEA<.06, CFI>.95), indicating strong measurement invariance for most pairfam scales; Anchor Questionnaire; Items:igr10;igr12;igr40p1;igr40p3	Brüderl, J, Hank K, Huinink J, Nauck B, Neyer F, Walper S, Alt P, Borschel E, Buhr P, Castiglioni L, Fiedrich S, Finn C, Garrett M, Hajek K, Herzig M, Huyer-May B, Lenke R, Müller B, Peter T, Schmiedeberg C, Schütze P, Schumann N, Thönnissen C, Wetzel M & Wilhelm B. 2017. The German Family Panel (pairfam). GESIS Data Archive, Cologne. ZA5678 Data file Version 8.0.0, doi: 10.4232/pairfam.5678.8.0.0.

			;igr94	
		Number of siblings	N.A.	Windzio, M & Fornahl,D , 2016: The role of school-related social networks for school leaving examination and career choice in a social and ethnical heterogenous group of pupils. GESIS Data Archive, Cologne. ZA6254 Data file Version 1.0.0, doi:10.4232/1.12476
	**Familial shaping**	Familial eating habits	N.A.	Warschburger P, Ruzanska U, 2017, Universität Potsdam (Self-constructed items)
		Familial attitudes	- Cronbach's alpha = .77 - .86<br>- Test–retest reliability: r = .51<br>- Convergent validity: Attitudes are associated with healthy eating behaviour, fruit and vegetable intake, behavioural intention, PBC, perceived past behaviour and subjective norms	Conner M, Norman P, Bell R. The theory of planned behavior and healthy eating. Health Psychology. 2002;21:194–201. doi:10.1037//0278-6133.21.2.194.<br><br>Bruijn G-J de, Kremers SPJ, Vet E de, Nooijer J de, van Mechelen W, Brug J. Does habit strength moderate the intention–behaviour relationship in the Theory of Planned Behaviour?: The case of fruit consumption. Psychology & Health. 2007;22:899–916. doi:10.1080/14768320601176113.
**OUTCOME**				
	**Dietary intake**	Food Frequency Questionnaire	- Use of fitted portion sizes did not clearly lead to improvements<br>- r = .57 and .58 for fitted and predefined portion sizes in men; r= .55 for both approaches to portion sizes in women	Nöthlings U, Hoffmann K, Bergmann MM, Boeing H: Fitting Portion Sizes in a Self-Administered Food Frequency Questionnaire. Journal of Nutrition, 2007. 137:2781–2786

		Short food list	- At least 80% reported good or very good perceived completeness of the list of food items and confirmed that the questionnaire reflected their diet of the previous 24 h.	Freese J, Pricop-Jeckstadt M, Heuer T, Clemens M, Boeing H, Knüppel S, Nöthlings U. Development and evaluation of a short 24-h food list as part of a blended dietary assessment strategy in large-scale cohort studies. Eur J Clin Nutr. 2014; 68:324-9. doi: 10.1038/ejcn.2013.274

Additional file Table S1: Detailed description including reliability and validity of the instruments and scales implemented in the NutriAct Family Study on Determinants of Food Choice (NFS);
